# Detection of diverse *Wolbachia *16S rRNA sequences at low titers from malaria vectors in Kayin state, Myanmar

**DOI:** 10.12688/wellcomeopenres.15005.4

**Published:** 2019-11-05

**Authors:** Sunisa Sawasdichai, Victor Chaumeau, Tee Dah, Thithiworada Kulabkeeree, Ladda Kajeechiwa, Monthicha Phanaphadungtham, Muesuwa Trakoolchengkaew, Praphan Kittiphanakun, Yanada Akararungrot, Kyi Oo, Gilles Delmas, Nicholas J. White, François H. Nosten

**Affiliations:** 1 Shoklo Malaria Research Unit, Mahidol-Oxford Tropical Medicine Research Unit, Faculty of Tropical Medicine, Mahidol University, Mae Sot, 63110, Thailand; 2 Centre for Tropical Medicine and Global Health, Nuffield Department of Medicine, University of Oxford, Oxford, OX3 7BN, UK; 3 Mahidol-Oxford Tropical Medicine Research Unit, Faculty of Tropical Medicine, Mahidol University, Bangkok, 10400, Thailand

**Keywords:** Wolbachia, Anopheles, Plasmodium, 16S rRNA, entomological inoculation rate, Southeast Asia, Kayin state, wAnga

## Abstract

**Background** **:** Natural *Wolbachia *infections in malaria mosquitoes were recently reported in Africa, and negatively correlated with the development of *Plasmodium falciparum* in the vectors. The occurrence and effects of *Wolbachia *infections outside Africa have not been described and may have been underestimated.

**Methods** **:** Mosquitoes were collected by human-landing catch during May and June 2017 in ten villages in Kayin state, Myanmar. Closely related species of malaria vectors were identified with molecular assays. 16S rRNA *Wolbachia* DNA sequences were detected with quantitative real-time PCR.

**Results:** Low titer of *Wolbachia *DNA was detected in 13/370 samples in six malaria vector species. Sequences were diverse and different from those described in the African malaria mosquitoes.

**Conclusion:** The detection of *Wolbachia* DNA in malaria mosquitoes from Kayin state warrants further investigations to understand better the ecology and biology of *Anopheles*- *Wolbachia* interactions in Southeast Asia.

## Introduction

*Wolbachia* are intracellular bacteria that infect a wide variety of arthropods and filarial nematodes. Symbiotic relationship that results from the infection have a broad range of phenotypic effects on the infected hosts, from mutualism (beneficial) to commensalism (neutral) and parasitism (harmful) ^1^. In mosquitoes, *Wolbachia* can invade the germline and induce cytoplasmic incompatibilities between the sperm from infected males and oocytes from uninfected females ^2^. Hence, mass-releases of *Wolbachia*-infected male mosquitoes were attempted to extinguish mosquito populations ^3, 4^. Cytoplasmic incompatibilities produce a fitness advantage of *Wolbachia*-infected over uninfected female mosquitoes, thereby driving the spread of *Wolbachia*-infected females in the population. In addition, *Wolbachia* can interfere with the development of some pathogens in the mosquito host, including dengue virus ^5^, *Plasmodium* malaria parasites ^6^ and filarial nematodes ^7^. Therefore, the release of *Wolbachia*-infected female mosquitoes is proposed for transmission-blocking of some mosquito-borne diseases ^8^.

Most diversions of mosquito- *Wolbachia* interactions for controlling vector-borne diseases were conducted with mosquitoes artificially infected with the endosymbiont. Natural *Wolbachia* infections may have important effects on mosquito populations and dynamics of diseases transmission but they are less well described ^9^. *Wolbachia* DNA was detected by PCR in 27 mosquito genera including the medically important *Aedes*, *Armigeres*, *Culex*, *Mansonia* and *Stegomiya* ^9– 18^. Interestingly, this organism was not detected in malaria mosquitoes until recent observations of naturally infected anopheline vectors in Africa ^9, 13, 19– 24^.

Only one study assessed the effects of natural *Wolbachia* infection on the reproductive fitness anopheline mosquitoes, namely the dominant African malaria vector *Anopheles coluzzi* ^22^. The authors did not observe cytoplasmic incompatibilities, differences in the number of eggs laid or progeny sex ratio, but infected females laid eggs more rapidly. Two studies demonstrated the negative effects of *Wolbachia* infections on the development of *P. falciparum* ^20, 22^. Shaw *et al.* observed a negative correlation between *Wolbachia* infection and the development of *P. falciparum* in naturally blood-fed females. Gomes *et al*. obtained similar results on the sporozoite stage by screening large numbers of mosquitoes identified as *An. gambiae sensu stricto* and *An. coluzzi*. In addition to their field investigations, Gomes *et al.* infected a laboratory-adapted *An. coluzzi* colony with a local strain of *Wolbachia*, and performed artificial transmission studies with cultured gametocytes of *P. falciparum* strain NF54. They observed a moderate yet significant positive correlation between *Wolbachia* infection and oocyst development, and a negative correlation between *Wolbachia* infection and the number of sporozoites that subsequently invaded the salivary glands.

Natural *Wolbachia* infections in Southeast Asian malaria vectors have not been reported. Their potential effects on *Anopheles* mosquitoes and dynamics of malaria transmission are not known. The objective of this study was therefore to assess the presence of *Wolbachia* in malaria vector populations in Kayin state, Myanmar.

## Methods

### Study sites and entomological collections

**Figure 1.**
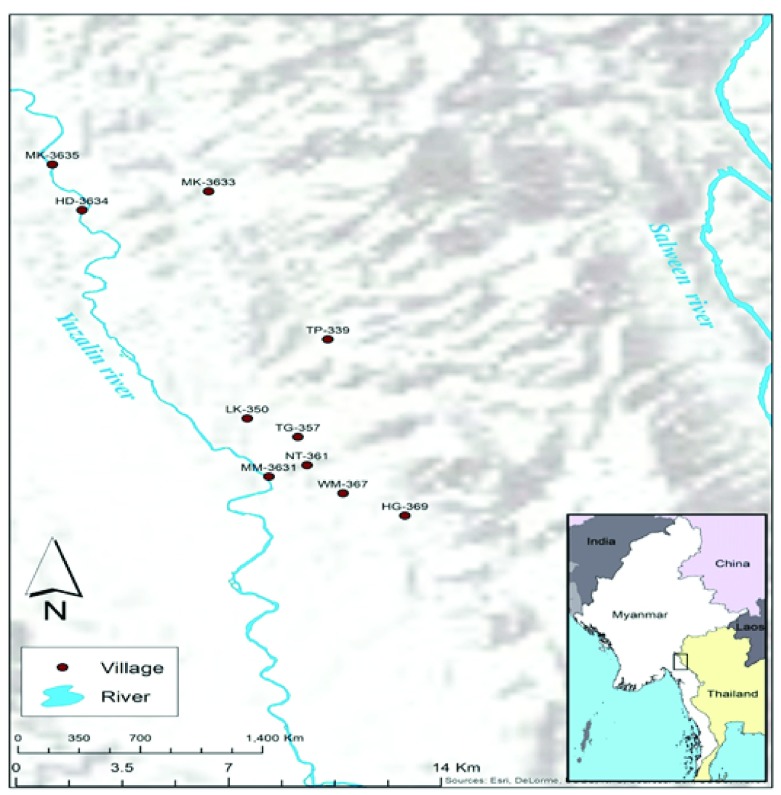
Map of the study area.

Entomological surveys were conducted in May and June 2017 in ten villages in Kayin state, Myanmar ( Figure 1). Each survey consisted of five consecutive nights of collection from 06:00 pm to 06:00 am as described previously ^25^. In each village, five traditional houses were selected for mosquito sampling with human-landing catches. Collectors were asked to collect every mosquitoes landing on their uncovered legs for 50 min per hour and allowed to rest for 10 min per hour. Mosquitoes were shipped to Mae Sot (Thailand) at the end of each survey.

### Malaria vectors identification

Mosquitoes were immediately identified at the genus level by morphology and *Anopheles* specimen were stored individually at -20°C in 1.5 mL plastic tubes containing silica gel. *Anopheles* were identified at the Group or Complex level using the key developed by Rattanarithikul *et al.* ^26^. Closely related species in the Funestus, Maculatus and Leucopshyrus Groups were discriminated in a subsample of the total number of collected mosquito using allele-specific PCR assays (AS-PCR) adapted from Garros *et al.* and Walton *et al.* ^27– 29^. Single whole mosquitoes were crushed in 200 μl of cetyl-trimethylammonium bromide solution 2% (TrisHCl pH = 8, 20mM; EDTA 10mM; NaCl, 1.4 mM; N-cetyl-N,N,N-trimethyl ammonium bromide 2%) with a TissueLyser II™ (Qiagen) set on 29 movements /second for 3 minutes. Samples were then warmed at 65°C for 5 minutes and 200 μl of chloroform were added. The aqueous phase was collected and DNA was precipitated with 200 μl of isopropanol. After centrifugation at 20,000 g for 15 minutes, the pellet was washed twice with 200 µl of 70% ethanol and suspended in 50 μl of PCR grade water ^30^. The PCR mix was composed of 1X Goldstar™ DNA polymerase (Eurogentec, Seraing, Belgium) and 400 nM of each primer (Funestus assay: ITS2A 5’-TGT GAA CTG CAG GAC ACA T-3’, MIA 5’-CCC GTG CGA CTT GAC GA-3’, MIC 5’-GTT CAT TCA GCA ACA TCA GT-3’, ACO 5’-ACA GCG TGT ACG TCC AGT-3’, PAM 5’-TGT ACA TCG GCC GGG GTA-3’, VAR 5’-TTG ACC ACT TTC GAC GCA-3’; Maculatus assay: 5.8F 5’-TGT GAA CTG CAG GAC ACA T-3’, MAC 5’-CCC GTG CGA CTT GAC GA-3’, PSEU 5’-GTT CAT TCA GCA ACA TCA GT-3’, SAW 5’-ACA GCG TGT ACG TCC AGT-3’, K 5’-TGT ACA TCG GCC GGG GTA-3’, DRAV 5’-TTG ACC ACT TTC GAC GCA-3’ and Leucopshyrus assay: D-AC 5’-CAC AGC GAC TCC ACA CG-3’, D-B 5’-CGG GAT ATG GGT CGG CC-3’, D-D 5’-GCG CGG GAC CGT CCG TT-3’, D-F 5’-AAC GGC GGT CCC CTT TG-3’, D-AC 5’-CAC AGC GAC TCC ACA CG-3’). The PCR was conducted in a total reaction volume of 25 μl (1 μl of DNA template and 24 μl of PCR mix). The thermocycling protocol consisted in an initial activation step of 1 minute at 94°C, followed by 40 amplification cycles of 20 seconds at 94°C, 20 seconds at the appropriate annealing temperature (45°C for the Funestus assay, and 55°C for the Maculatus and Leucosphyrus assays), and 30 seconds at 72°C. The length of the PCR product was determined by gel electrophoresis in 2% agarose for 70 minutes at 120V. In case AS-PCR gave a negative result, amplification of ITS2 was performed using the primer pair ITS2A (5'-TGT GAA CTG CAG GAC ACA T-3') and ITS2B (5'-ATG CTT AAA TTY AGG GGG T-3') described by Beebe and Saul ^31^. The PCR mix was composed of 1X Goldstar™ DNA polymerase (Eurogentec, Seraing, Belgium) and 400 nM of each primer. The PCR was conducted in a total reaction volume of 25 μl (1 μl of DNA template and 24 μl of PCR mix). The thermocycling protocol consisted in an initial activation step of 1 minute at 94°C, followed by 40 amplification cycles of 20 seconds at 94°C, 20 seconds at 51°C and 30 seconds at 72°C. PCR products were purified on site using the Illustra™ ExoStar™ PCR and Sequence Reaction Clean-Up Kit (GE Healthcare) according to manufacturer’s instruction. Macrogen (Seoul, South Korea) sequenced the purified PCR products off site with the ITS2A primer. Sequences were blasted against the National Center for Biotechnology Information nucleotide database in order to determine the corresponding species (accession numbers MK358471 - MK358807).

### Detection of *Wolbachia* DNA by quantitative real-time PCR

Two primer sets were considered for *Wolbachia* screening in mosquito samples: W-Specf/W-Specr (5’-CAT ACC TAT TCG AAG GGA TAG-3’ and 5’- AGC TTC GAG TGA AAC CAA TTC-3’) amplified a 438 bp conserved region of the 16S rRNA genes and W-Specf/W16S (5’- CAT ACC TAT TCG AAG GGA TAG -3’ and 5’- TTG CGG GAC TTA ACC CAA CA -3’) amplified a shorter fragment of the same region (102 bp). These two sets were selected because they were previously used by other in order to detect *Wolbachia* in *Anopheles* mosquitoes ^20^. Without *a priori* knowledge on *Wolbachia* DNA sequences detected in this study, the W-Specf/W-Specr primer set was selected for its ability to detect most *Wolbachia* strains infecting insects and to establish phylogenetic relationships among isolates ^32^.

The performances of the primers W-Specf/W-Specr for the detection and quantitation of *Wolbachia* in mosquito samples were compared to that of the primers W-Specf/W16S as described previously ^30^. Briefly, a published strain of laboratory-reared *Aedes aegypti* artificially infected with *Wolbachia* strain wMel were used as a reference material ^33^. The optimal conditions for the PCR (hybridization temperature for primers annealing, and concentration of MgCl _2_ and primers) were determined during a single gradient experiment in order to take into account cross-interactions between the different parameters. The range tested were 55–62°C for the hybridization temperature, 2.5–4.5 mM of MgCl _2_ and 100–400 nM of each primers. The reaction conditions that gave the smallest CP (optimal conditions) were selected for all subsequent experiments. Serial-dilution experiments were then carried out in order to verify PCR efficiency (EFF) and to estimate the standard curve parameters.

All experiments were conducted with a CFX-96® (Biorad) device. Reactions were conducted in 20µl of EVAGreen qPCR Mix Plus® (Euromedex); 5µl of DNA template was used in a total reaction volume of 25µl. The PCR mix was composed of 1X HOT FIREPol™ EvaGreen™ qPCR Mix Plus (Solis BioDyne, Tartu, Estonia) and 200 nM of each primer. The thermocycling protocol consisted in an activation step at 95°C for 15 minutes followed by 45 amplification cycles at 95°C for 15 seconds, 58°C for 15 seconds and 72°C for 20 seconds. PCR products were characterized by analyzing amplicon melt curve (95°C for 15 seconds, 68°C for 1 minute, 80°C for 15 seconds, 60°C for 15 seconds, then 60°C to 90°C with an increment of 0.2°C per second). No template and positive controls were included in all runs. All samples and controls were tested in triplicates.

Specificity of the PCR was confirmed by Sanger sequencing with both W-Specf/W-Specr primers for all samples that give at least 1/3 positive reaction. Positive reaction was defined by the presence of a PCR product with the same melting temperature than the positive control at the end of the thermocycling. Macrogen (Seoul, South Korea) performed both PCR product purification and sequencing off site to avoid contamination of our facilities with post-PCR amplicons. The sequences were used for phylogenetic analysis (accession numbers MK336794 - MK336806).

### Data analysis

Human-biting rate was defined as the number of collected mosquitoes divided by the corresponding number of collection-nights. Poisson confidence intervals were calculated using the *epitools* package version 0.5–10 in R software. Human-biting rate for *sensu stricto* species in the Funestus, Maculatus and Leucopshyrus Groups was estimated using the relative proportion of the species in the corresponding group.

The limit of detection of the qPCR assay (LOD) was defined as the highest dilution (lowest concentration) that gave 100% of positive reactions. The performances of the two primer sets at low concentrations of *Wolbachia* were also compared by scoring the proportion of positive reactions as described previously ^30, 34^. Crossing-point (CP) values were determined using the regression algorithm of the analysis software of the PCR device (CFX Biorad Manager version 3.01, Biorad). CP values of standard samples in the serial-dilution experiments were used to set-up the standard curve of the assay. The best fit-line and the subsequent values of the slope and y-intercept were estimated by performing least-square analysis of the linear portion of the curve (Pearson’s coefficient r ^2^>0.990). PCR efficiency was estimated with the formula EFF = 10 ^(-1/slope)^–1.

For the phylogenetic analysis, chimeric PCR products were detected with the DECIPHER software version 2.0 and excluded from subsequent analysis (4/17 samples with a positive PCR result). 16S ssuRNA sequences were blasted against the National Center for Biotechnology Information nucleotide database and the most similar sequence was downloaded. Reference *Rickettsiales* sequences were added and alignment was performed using the DECIPHER package version 2.10 in R software. DNA sequences were converted into RNA sequences and then aligned using the AlignSeqs() function set with default parameters in order to take into account base pairing and to use single-base and double-base substitution matrices. Tamura-Nei genetic distance model and neighbor-joining tree were computed with the *ape* package version 5.2 of the R software. There was 373 positions in the final dataset.

### Ethical considerations

This project was approved through the ethics review committee on medical research involving human beings from Myanmar, Ministry of Health and Sports, Department of Medical Research (lower Myanmar): 73/Ethics 2014. All participants provided their written consent to participate in this study.

## Results

### qPCR assay validation for the detection of *Wolbachia* in mosquitoes

**Figure 2.**
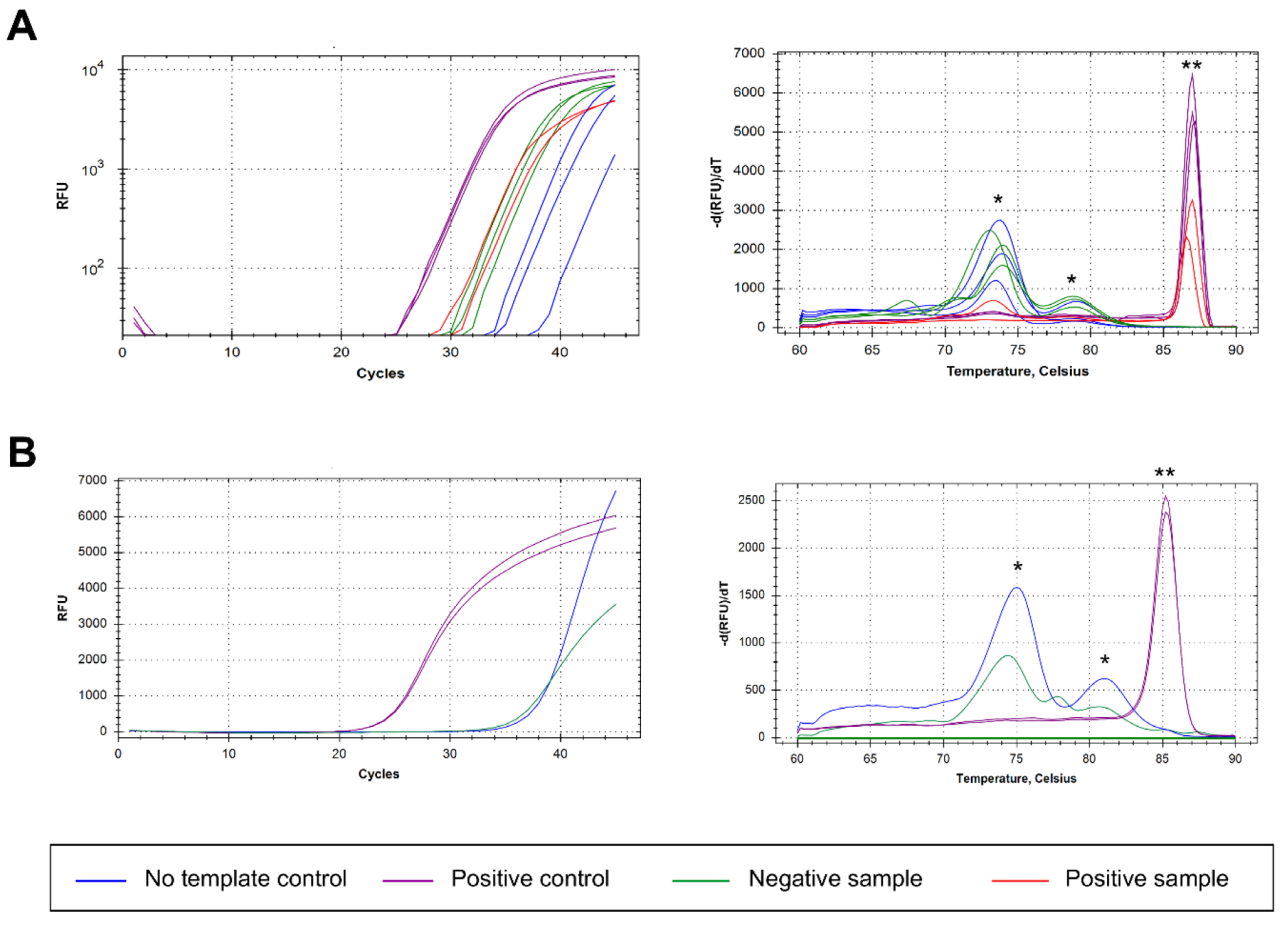
Typical result of the qPCR assay used for *Wolbachia* detection in mosquito samples. **A**) W-Specf/W-Specr primers; **B**) W-Specf/W16S primers. Left panels show amplification curves and right panels show the melt curve of the PCR products. (*) primer dimers, (**) PCR DNA target.

Optimal reaction conditions were similar for both primer sets: 58°C for primer annealing (range tested= 55–62°C), 2.5 mM of MgCl _2_ (range tested= 2.5–4.5 mM) and 200 nM of each primers (range tested= 100–400 nM). In these conditions, PCR efficiency was 108 and 110% with the primer sets W-Specf/W-Specr and W-Specf/W16S respectively, and the linear dynamic spanned over six orders of magnitude ( *r²*=0.998 and 0.999) ( Table 1). There was a one-log decrease in the LOD of the assay when using W16S as a reverse primer instead of W-Specr, and the assay scored better at low concentrations of *Wolbachia* (16/18 and 12/18 positive reactions respectively, χ ^2^= 2.5714, *P*=0.109). Typical amplification and melt curves are shown in the Figure 2.

**Table 1.** Results of the serial dilution experiments.

Primers (%EFF, *r* ^2^) ^a^	Parameter	Value of the parameter at the indicated dilution								Score (%) ^e^
		Not diluted	10 ^-1^	10 ^-2^	10 ^-3^	10 ^-4^	10 ^-5^	10 ^-6^	10 ^-7^	
**W-Specf/W-Specr** **(108%, 0.998)**	Nb. pos. / Nb. tested ^b^	**9/9**	**9/9**	**9/9**	**9/9**	**9/9**	**7/9**	5/9	0/9	12/18 (66%)
	Mean CP value	**18.81**	**21.26**	**24.68**	**28.01**	**30.73**	**34.48**	34.95	-	
	Intra-assay SD ^c^	**0.07**	**0.04**	**0.03**	**0.10**	**0.25**	**1.64**	0.33	-	
	Inter-assay SD ^d^	**0.11**	**0.03**	**0.02**	**0.09**	**0.25**	**1.45**	0.71	-	
**W-Specf/W16S** **(110%, 0.999)**	Nb. pos. / Nb. tested	**9/9**	**9/9**	**9/9**	**9/9**	**9/9**	**9/9**	7/9	0/9	16/18 (88%)
	Mean CP value	**17.80**	**20.71**	**23.98**	**27.29**	**29.90**	**33.40**	34.86	-	
	Intra-assay SD	**0.05**	**0.03**	**0.02**	**0.10**	**0.24**	**0.65**	1.01	-	
	Inter-assay SD	**0.01**	**0.06**	**0.14**	**0.18**	**0.09**	**0.10**	1.26	-	

^a^ %EFF : efficiency (EFF) of the PCR was calculated with the formula EFF = 10 ^(-1/slope)^ - 1 and expressed as a percentage. An efficiency of 100% corresponds to a slope of -3.32 and means that the number of amplicons doubles after each cycle of amplification; r ^2^: Pearson’s correlation coefficient expressing the intensity of the relationship between the logarithm of the concentration and the mean CP value. r ^2^ varies between 0 (no correlation) and 1 (perfect correlation), a value >0.990 testify of the linearity of the method (over a defined linear range) and allow an accurate quantification. r ^2^ and EFF have been calculated on the linear dynamic of each curve (bold cells). ^b^ Nb. pos. / Nb. tested: number of positive reactions (amplification of the PCR DNA target) / total of reactions performed at a given dilution. ^c^ Intra-assay SD : intra-assay standard deviation (SD), calculated as the average SD of the mean CP value measured for each dilution during the same experiment. ^d^ Inter-assay SD : inter-assay standard deviation (SD), calculated as the SD of the means CP values measured during two independent experiments. ^e^ score of the proportion of positive reactions at low concentrations of *Wolbachia* (score was calculated on dilutions 10 ^-5^ and 10 ^-6^); an example of the calculation of the score is given here : the maximum hit for the score is 18 reactions (9 at the dilution 10 ^-5^, +9 at the dilution 10 ^-6^), the score obtained with the primer pair W-Specf/W-Specr is 66% (12/18=(7+5)/18).

### Biodiversity of *Anopheles* mosquitoes

Four thousand seven hundreds forty-three *Anopheles* were collected during 500 person-nights of collection. We report the occurrence of 12 *Anopheles* taxa among which nine were groups of closely related or sibling species (Maculatus, Funestus, Jamesii, Leucosphyrus, Annularis, Barbirostris, Subpictus, Hyrcanus and Asiaticus Groups) and only three were *sensu stricto* species ( *An. karwari*, *An. kochi* and *An. tessellatus*). A subsample of 1098 mosquitoes in the Maculatus, Funestus and Leucosphyrus Groups were identified at the species level with molecular assays. The most frequently detected species were *An. maculatus* ( *s.s.*), *An. sawadwongporni* and *An. pseudowillmori* in the Maculatus Group, *An. minimus* ( *s.s.*), *An. culicifacies* B and *An. jeyporiensis* in the Funestus Group and *An. baimaii* in the Leucosphyrus Group ( Table 2).

**Table 2.** Village-collated human-biting rate estimates of *Anopheles* mosquitoes.

Group	Species	Human-biting rate estimate (95%CI) in the indicated dilution, expressed in number of bites/person/month									
		HD-3634	HG-369	LK-350	MK-3633	MK-3635	MM-3631	NT-361	TG-357	TP-339	WM-367
Annularis	*An. annularis* ( *s.l.*) ^b^	40.2 (31.2-51.1)	28.8 (21.2-38.2)	24 (17.1-32.7)	29.4 (21.8-38.9)	4.2 (1.7-8.7)	30 (22.3-39.6)	40.2 (31.2-51.1)	65.4 (53.7-78.9)	7.2 (3.7-12.6)	28.8 (21.2-38.2)
Asiaticus	*An. asiaticus* ( *s.l.*)	0 (0-2.2)	0 (0-2.2)	0 (0-2.2)	0 (0-2.2)	0 (0-2.2)	0 (0-2.2)	0 (0-2.2)	0 (0-2.2)	0.6 (0-3.3)	0 (0-2.2)
Barbirostris	*An. barbirostris* ( *s.l.*) ^b^	27 (19.7-36.1)	2.4 (0.7-6.1)	16.8 (11.2-24.3)	3.6 (1.3-7.8)	5.4 (2.5-10.3)	10.2 (5.9-16.3)	3.6 (1.3-7.8)	24.6 (17.7-33.4)	6 (2.9-11)	2.4 (0.7-6.1)
Funestus	*An. aconitus* ^b^	3 (1-7)	1.2 (0.1-4.3)	9.6 (5.5-15.6)	6 (2.9-11)	3.6 (1.3-7.8)	0 (0-2.2)	0 (0-2.2)	0 (0-2.2)	0 (0-2.2)	0 (0-2.2)
	*An. culicifacies* A ^c^	9 (5-14.8)	0 (0-2.2)	0 (0-2.2)	0 (0-2.2)	0 (0-2.2)	3.6 (1.3-7.8)	6 (2.9-11)	4.2 (1.7-8.7)	1.8 (0.4-5.3)	0 (0-2.2)
	*An. culicifacies* B	36.6 (28-47)	7.2 (3.7-12.6)	0 (0-2.2)	73.8 (61.3-88.1)	12.6 (7.8-19.3)	123.6 (107.3-141.7)	94.2 (80-110.1)	7.8 (4.2-13.3)	1.8 (0.4-5.3)	26.4 (19.2-35.4)
	*An. harrisoni*	3 (1-7)	0 (0-2.2)	0 (0-2.2)	0 (0-2.2)	0 (0-2.2)	0 (0-2.2)	0 (0-2.2)	4.2 (1.7-8.7)	0 (0-2.2)	0.6 (0-3.3)
	*An. jeyporiensis* ^c^	3 (1-7)	1.2 (0.1-4.3)	12.6 (7.8-19.3)	6 (2.9-11)	44.4 (34.9-55.7)	3.6 (1.3-7.8)	3 (1-7)	78.6 (65.7-93.3)	3.6 (1.3-7.8)	0 (0-2.2)
	*An. minimus* ^a^	161.4 (142.7-181.9)	46.8 (37-58.4)	240 (217.1-264.7)	185.4 (165.3-207.3)	96 (81.7-112.1)	167.4 (148.3-188.2)	141.6 (124.1-160.9)	259.2 (235.3-284.8)	141 (123.5-160.2)	52.2 (41.8-64.4)
	*An. varuna*	0 (0-2.2)	0 (0-2.2)	0 (0-2.2)	0 (0-2.2)	0 (0-2.2)	0 (0-2.2)	0 (0-2.2)	4.2 (1.7-8.7)	0 (0-2.2)	0 (0-2.2)
Hyrcanus	*An. hyrcanus* ( *s.l.*) ^c^	0.6 (0-3.3)	0.6 (0-3.3)	2.4 (0.7-6.1)	0.6 (0-3.3)	0 (0-2.2)	0 (0-2.2)	2.4 (0.7-6.1)	0.6 (0-3.3)	0 (0-2.2)	0 (0-2.2)
Jamesii	*An. jamesii* ( *s.l.*) ^c^	140.4 (123-159.6)	27 (19.7-36.1)	9 (5-14.8)	15.6 (10.2-22.9)	8.4 (4.6-14.1)	15.6 (10.2-22.9)	46.2 (36.5-57.7)	141.6 (124.1-160.9)	119.4 (103.4-137.2)	44.4 (34.9-55.7)
Kochi	*An. kochi*	37.8 (29-48.4)	1.8 (0.4-5.3)	3 (1-7)	0.6 (0-3.3)	55.8 (45-68.4)	6.6 (3.3-11.8)	15.6 (10.2-22.9)	36 (27.5-46.3)	54 (43.4-66.4)	2.4 (0.7-6.1)
Leucosphyrus	*An. baimaii* ^a^	31.8 (23.8-41.6)	1.8 (0.4-5.3)	24.6 (17.7-33.4)	10.2 (5.9-16.3)	13.2 (8.3-20)	18.6 (12.6-26.4)	15 (9.7-22.1)	24 (17.1-32.7)	11.4 (6.9-17.8)	6.6 (3.3-11.8)
	*An. dirus* ^a^	2.4 (0.7-6.1)	0 (0-2.2)	0 (0-2.2)	1.2 (0.1-4.3)	0 (0-2.2)	1.8 (0.4-5.3)	0 (0-2.2)	1.8 (0.4-5.3)	0.6 (0-3.3)	0 (0-2.2)
	*An. introlatus*	0 (0-2.2)	0 (0-2.2)	0 (0-2.2)	0 (0-2.2)	0 (0-2.2)	0 (0-2.2)	0 (0-2.2)	0 (0-2.2)	0.6 (0-3.3)	0 (0-2.2)
Maculatus	*An. maculatus* ^a^	241.2 (218.2-266)	40.8 (31.7-51.7)	88.2 (74.5-103.7)	26.4 (19.2-35.4)	59.4 (48.3-72.3)	61.8 (50.4-75)	226.8 (204.5-250.9)	1112.4 (1062.3- 1164.2)	315 (288.6-343.1)	39 (30.1-49.7)
	*An.* *pseudowillmori* ^b^	6.6 (3.3-11.8)	3.6 (1.3-7.8)	0 (0-2.2)	4.2 (1.7-8.7)	15 (9.7-22.1)	1.2 (0.1-4.3)	3.6 (1.3-7.8)	19.2 (13.1-27.1)	0 (0-2.2)	0 (0-2.2)
	*An. rampae*	0 (0-2.2)	0 (0-2.2)	0 (0-2.2)	0.6 (0-3.3)	0 (0-2.2)	0 (0-2.2)	0 (0-2.2)	38.4 (29.6-49)	0 (0-2.2)	0 (0-2.2)
	*An.* *sawadwongporni* ^a^	42 (32.7-53.1)	12.6 (7.8-19.3)	25.2 (18.2-34.1)	21.6 (15.1-29.9)	3.6 (1.3-7.8)	16.8 (11.2-24.3)	66 (54.2-79.5)	307.2 (281.2-335)	42 (32.7-53.1)	12 (7.3-18.5)
Maculatus- related	*An. karwari* ^c^	15 (9.7-22.1)	2.4 (0.7-6.1)	0 (0-2.2)	2.4 (0.7-6.1)	1.2 (0.1-4.3)	0 (0-2.2)	3 (1-7)	11.4 (6.9-17.8)	3 (1-7)	0.6 (0-3.3)
Subpictus	*An. subpictus* ( *s.l.*) ^c^	79.8 (66.8-94.6)	40.8 (31.7-51.7)	41.4 (32.2-52.4)	13.2 (8.3-20)	5.4 (2.5-10.3)	58.8 (47.7-71.7)	137.4 (120.2-156.4)	24.6 (17.7-33.4)	182.4 (162.5-204.1)	119.4 (103.4-137.2)
Tessellatus	*An. tessellatus* ^c^	3.6 (1.3-7.8)	0 (0-2.2)	24 (17.1-32.7)	2.4 (0.7-6.1)	0 (0-2.2)	14.4 (9.2-21.4)	8.4 (4.6-14.1)	1.2 (0.1-4.3)	3.6 (1.3-7.8)	0.6 (0-3.3)

^a^ primary malaria vectors; ^b^ secondary malaria vectors, ^c^ efficient malaria vector species in some areas that were never reported infected with human malaria parasites on the Thailand-Myanmar border ^25^. Human-biting rates of *sensu stricto* anopheline species in the Funestus, Maculatus and Leucosphyrus Groups were estimates from the relative proportion of each species in the corresponding Group assessed with molecular assays.

### Detection of *Wolbachia* DNA in malaria vectors

**Figure 3.**
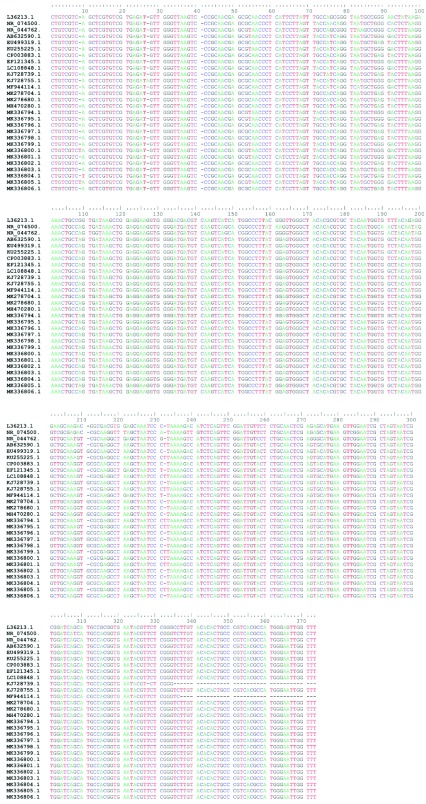
Multiple alignment of 16S RNA sequences used to build the Tamura-Nei genetic distance model and neighbor-joining tree.

**Figure 4.**
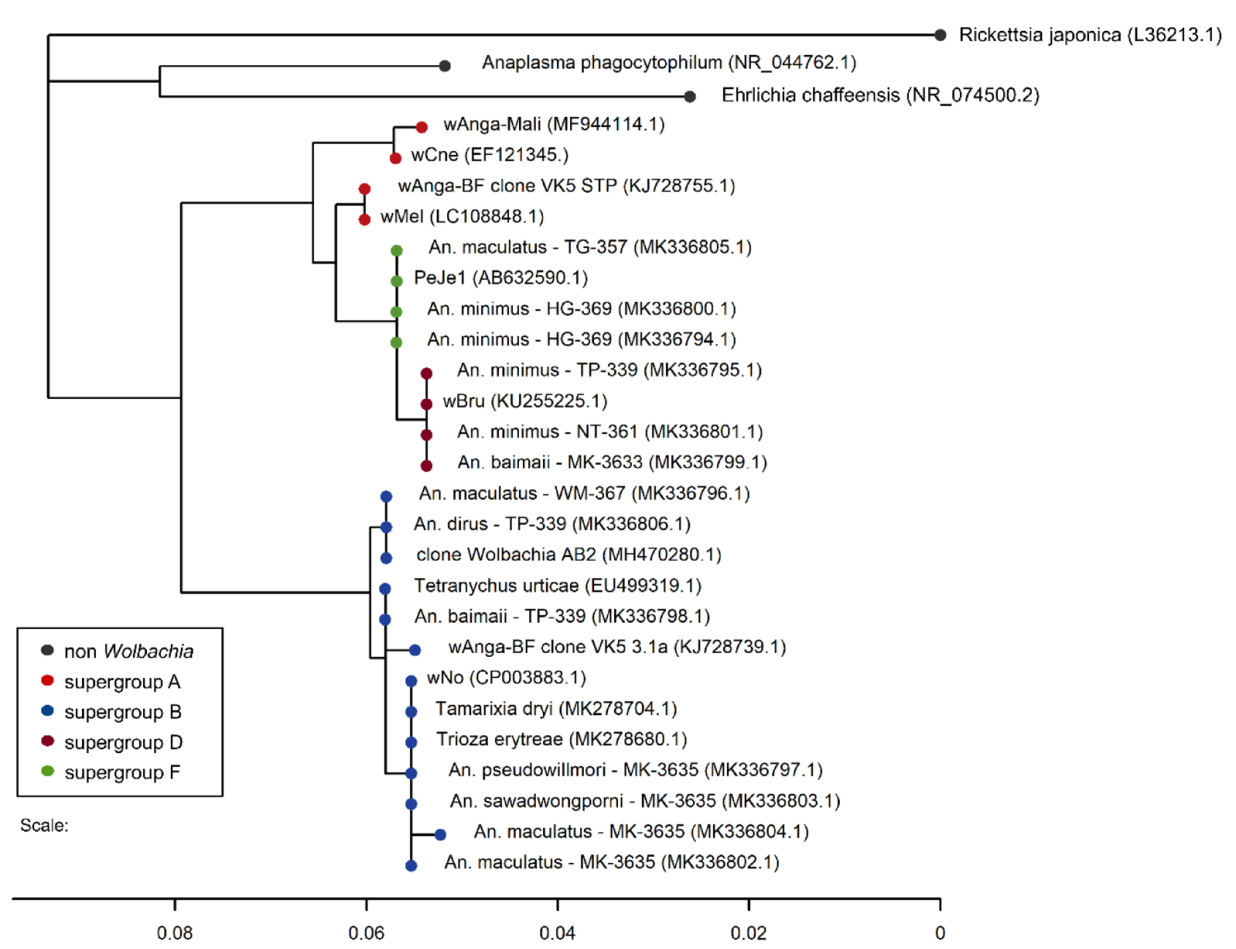
Phylogenetic analysis based on the alignment of a conserved region of the 16S rRNA gene using *Wolbachia*-specific primer pair W-Specf/W-Specr. Sequences of the PCR products were blasted against the NCBI nucleotide database and the most similar result was downloaded. A phylogenetic tree was reconstructed using a Tamura-Nei genetic distance model and neighbor joining. Sequences from other non- *Wolbachia* proteobacteria were also included, and the sequence from *Rickettsia japonica* was used as the reference outgroup. There was 373 positions in the final dataset. Nodes with bootstrap support <50% were collapsed. Study samples were labeled with the host name and the study village, and the accession number reported into the brackets. Formally named *Wolbachia* strains were labeled with their abbreviation: wNo is a symbiont of *Drosophila simulans*, wCne of *Ctenocephalides felis*, wAlbB of *Aedes albopictus*, wAnga of *An. gambiae*, wMel of *Drosophila melanogaster*, wPeJe1 of *Penicillidia jenynsii* and wBru of *Brugia malayi*.

The presence of *Wolbachia* DNA was assessed in six *Anopheles* species namely *An. maculatus* ( *s.s.*), *An. sawadwongporni*, *An. pseudowillmori* (Maculatus Group), *An. minimus* ( *s.s.*) (Funestus Group, Minimus Complex), *An. dirus* ( *s.s.*) and *An. baimaii* (Leucosphyrus Group, Dirus Complex). *Wolbachia* DNA was detected in 13/370 samples ( Table 3). Eight unique 16S rRNA sequences were identified ( Figure 3). 16S rRNA sequences clustered with that of *Wolbachia* strains in the supergroups B, D and F ( Figure 4). 

**Table 3.** Results of the screening for natural *Wolbachia* infections in the ten villages.

Group	Species	Nb. pos / Nb. tested ( *Wolbachia* supergroup) in the indicated village and species									
		HD-3634	HG-369	LK-350	MK-3633	MK-3635	MM-3631	NT-361	TG-357	TP-339	WM-367
Funestus	*An. minimus*	0/10	2/10 (F)	0/10		0/10	0/10	1/10 (D)	0/10	1/10 (D)	0/10
Maculatus	*An. maculatus*	0/10	0/10	0/10		2/10 (B)	0/10	0/10	1/10 (F)	0/11	1/9 (B)
	*An. pseudowillmori*	0/1	0/1		0/1	1/7 (B)			0/1		
	*An. sawadwongporni*	0/8	0/3	0/9	0/10	1/1 (B)	0/10	0/11	0/10	0/4	0/2
Leucopshyrus	*An. baimaii*	0/10	0/2	0/10	1/10 (D)	0/11	0/10	0/10	0/10	1/16 (B)	0/10
	*An. dirus*	0/4			0/2		0/2		0/3	1/1 (B)	

Crossing-point values ranged from 31.0 to 40.6 amplification cycles and *Wolbachia* DNA titers were generally close or below the limit of detection of the assay (only one sample gave 3/3 positive reactions) ( Table 4).

**Table 4.** qPCR results of the *Wolbachia*-infected samples detected during the screening.

Sample ID	Village	Species	Nb pos	CP1	CP2	CP3	Supergroup
1	HG-369	*An. minimus*	1	35.8			F
2	HG-369	*An. minimus*	1	33.0			F
3	MK-3633	*An. baimaii*	1	35.6			D
4	MK-3635	*An. maculatus*	1	34.3			B
5	MK-3635	*An. maculatus*	1	34.3			B
6	MK-3635	*An. pseudowillmori*	1	37.6			B
7	MK-3635	*An. sawadwongporni*	2	34.5	32.8		B
8	NT-361	*An. minimus*	3	36.8	35.8	36.6	D
9	TG-357	*An. maculatus*	1	34.2			F
10	TP-339	*An. baimaii*	3	33.0	31.0	32.3	B
11	TP-339	*An. dirus*	1	34.1			B
12	TP-339	*An. minimus*	1	40.6			D
13	WM-367	*An. maculatus*	1	32.6			B

## Discussion

*Wolbachia* DNA was detected for the first time in Southeast Asian malaria vectors, including *An. maculatus* ( *s.s.*), *An. sawadwongporni*, *An. pseudowillmori* (Maculatus Group), *An*. *dirus* ( *s.s.*) and *An. baimaii* (Dirus Complex, Leucosphyrus Group).

CP values reported in this study suggest that *Wolbachia* DNA titers were very low, usually close or below the limit of detection of our assay. This result is not compatible with the integration of *Wolbachia* DNA in the mosquito genome, which would have given much lower CP values ^35^. Important precautions were taken to ensure the quality of our molecular data ^36^. This was the first study on *Wolbachia* in our facilities. The 16S DNA sequences detected in the screened samples were different from that of the reference material, hence excluding cross-contaminations. In addition, all experiments were conducted with the real-time PCR technology (which allows amplification and detection of the PCR DNA target in a closed system) and great care was taken to perform all handlings of PCR products off site. These precautions, combined with the good laboratory practices relevant to molecular diagnosis (eg. dedicated facilities with unidirectional workflow, experiment conducted by qualified laboratory technicians and appropriate quality controls), drastically limited the risk of false positive by contamination. The risk of false positive results due to low specificity of the assay was ruled out by sequencing the PCR product in all positive samples. It is probable that some results were falsely negative due to limited sensitivity, given that most positive samples were infected at a density close of below the detection of the assay. In this study, we have shown that using the W16S as a reverse primer increases the analytical sensitivity of the qPCR assay in the optimal reaction conditions. However, in the absence of *a priori* data on the *Wolbachia* DNA sequences detected in this study, we selected the W-Specf/W-Specr primers to perform the screening because of their availability to detect a wide variety of *Wolbachia* infecting insects and to establish phylogenetic relationships among field isolates ^32^. Molecular phylogeny based on 16S rRNA sequences revealed a high diversity of *Wolbachia* strains, which belonged to different lineages than those recently reported in the African malaria vectors ^19– 24^. Eight out of thirteen sequences reported in this study were unique. The DNA extracts were also used to assess *Plasmodium* infection rates in the mosquito population (data not shown), precluding multi locus sequence typing of the *Wolbachia* strains because there was no material remaining after the screening.

The significance of these findings regarding the biology and ecology of *Wolbachia-Anopheles* interactions must be interpreted cautiously as the detection of low titers of *Wolbachia* DNA by PCR is not unequivocal of an actual symbiosis between Wolbachia and the mosquito. The detection of *Wolbachia* in the supergroup D and F suggests that some DNA extracts were contaminated with *Wolbachia* endosymbionts of filarial nematodes rather than reflecting actual *Wolbachia* infections in mosquitoes. Chrostek and Gerth further argued that the high diversity of *Wolbachia* sequences combined with the very low titers detected was incompatible with the notion of a stable, intraovarially-transmitted *Wolbachia* symbiont in *An. gambiae* ^37^. Given that most arthropods are infected with *Wolbachia* ^38^, we cannot exclude that the DNA sequences detected in this study come from some sort of environmental contamination. An alternative explication could be that horizontal transfers of *Wolbachia* happen at a much higher frequency than previously thought, for example via plants ^39^ or via ectoparasitic mites ^40, 41^. Additional experiments would be of great interest to demonstrate actual infection, e.g. showing intracellular localization of the sequences and maternal transmission of the bacteria. Finally, we did not assess the effects of the presence of *Wolbachia* DNA on the phenotype of mosquitoes and dynamics of malaria transmission. In Kayin state, malaria transmission is low, seasonal and unstable. *Plasmodium* infection rate is usually less than 1% and often nil in the mosquito populations ^25^. Therefore, it was not possible to establish direct correlations between *Plasmodium* and the presence of *Wolbachia* DNA in the mosquito vectors. In this setting, the effect of possible *Wolbachia* infections on malaria transmission may be better assessed by performing artificial infections of field-collected mosquitoes with *Plasmodium* malaria parasites.

## Conclusion

The detection *Wolbachia* DNA in malaria vectors from Kayin state warrants further investigations to understand better the ecology and biology of *Anopheles*- *Wolbachia* interactions in Southeast Asia.

## Data availability

The data is available upon request to the Mahidol Oxford Tropical Medicine Research Unit Data Access Committee ( http://www.tropmedres.ac/data-sharing) and following the Mahidol Oxford Tropical Medicine Research Unit data access policy ( http://www.tropmedres.ac/_asset/file/data-sharing-policy-v1-0.pdf).
