# Supplementary material for: Detection of diverse Wolbachia 16S rRNA sequences at low titers from malaria vectors in Kayin state, Myanmar
Source: Wellcome Open Res. 2019 Nov 5;4:11. Originally published 2019 Jan 24. [Version 4] doi: 10.12688/wellcomeopenres.15005.4 (PMC6892426; doi:10.12688/wellcomeopenres.15005.4)
Supplement: Peer review history

# Peer review: Reviewer response for version 4

**DOI:** 10.21956/wellcomeopenres.17037.r36934

**Published:** 2019-12-03

**Authors:** Ewa Chrostek

**Affiliations:** 1 Institute of Integrative Biology, University of Liverpool, Liverpool, UK

Thank you for the revisions. The problematic "Wolbachia infection" has been mostly replaced, except from one place in the discussion. Also, I disagree with calling the Wolbachia DNA from previous reports an infection in the abstract and the introduction of this paper. In my opinion, this study is not defficient compared to the previous ones on Wolbachia in Anopheles. It only has the potential to be more carefully worded.

 I agree with the author's interpretation of the qPCR results. However, as primer dimers contribute to the total SYBR green fluorescence in the qPCR reaction this assay cannot be quantitative. Also, only running the qPCR products on the agarose gel can reveal if the bands can indeed be distinguished by a conventional PCR. As the authors do not aim at PCR-identification or qPCR quantification of Wolbachia here this is not crucial. It is curious though, and suggests that the qPCR assay could be optimized further.

I confirm that I have read this submission and believe that I have an appropriate level of expertise to confirm that it is of an acceptable scientific standard.

# Peer review: Reviewer response for version 3

**DOI:** 10.21956/wellcomeopenres.16939.r36677

**Published:** 2019-10-15

**Authors:** Ewa Chrostek

**Affiliations:** 1 Institute of Integrative Biology, University of Liverpool, Liverpool, UK

In this study, the authors provide evidence for qPCR amplification of a fragment of Wolbachia 16S sequence from 13 out of 370 field-collected Southeast-Asian mosquitoes. However, a single, low level, hard to amplify (despite extensive optimisation) fragment of Wolbachia rDNA does not provide sufficient evidence for the Wolbachia infection in these malaria vectors. The environment is severely contaminated with Wolbachia as most arthropods are infected with this symbiont (Weinert *et al*. 2015 ^1^). To show an infection, additional experiments are necessary, e.g. showing intracellular localization of the sequences and maternal transmission of the bacteria (see Chrostek and Gerth 2019 ^2^ for further discussion). I suggest changing the wording throughout, from “natural Wolbachia infections” to “Wolbachia 16S/wSpec sequence amplification”. I also recommend changing the title to: “Detection of low-density Wolbachia 16S sequences from malaria vectors in Kayin state, Myanmar”.

 In the results section, the authors identify the supergroups D and F Wolbachia sequences as likely environmental/parasitic contaminants (6 out of 13 positive samples). It is unclear why they do not use the same caution while interpreting data on the remaining 7 sequences from supergroup B.

 Figure 2 shows an interesting property of wSpec primer set. The amplification from the positive and negative samples produces similar amplification curves (Fig. 2A, green and red lines) with very similar CPs (crossing-points), that can be distinguished from each other by the shape of their melt curved (Fig. 2B). This figure shows, that when using wSpec primer set for a PCR, negative samples produce an amplicon as well. Whether the positive and negative amplicons can be distinguished by agarose gel electrophoresis, and therefore if classical PCR with wSpec has any diagnostic value when used under this conditions, remain to be determined. The comment on this in the text would help future studies embarking on Wolbachia identification projects.

 Minor comments:

1. The information on the origin of the laboratory-reared wMel-infected *A. aegypti* is missing. Were they made by the authors or are they the published strains?
2. The sequence of W16S primer, the amplicon sizes for both qPCR reactions and the rationale behind trying both primer sets should be added.
3. In the discussion, p7/28, first line: the integration into the chromosome would have produced “higher CP values”, and not as it is - “lower”.

I confirm that I have read this submission and believe that I have an appropriate level of expertise to state that I do not consider it to be of an acceptable scientific standard, for reasons outlined above.

## References

[1] : The incidence of bacterial endosymbionts in terrestrial arthropods. *Proc Biol Sci*.2015;282(1807) : 10.1098/rspb.2015.0249 20150249 10.1098/rspb.2015.0249 25904667PMC4424649

[2] : IsAnopheles gambiae a Natural Host ofWolbachia ?. *mBio*.2019;10(3) : 10.1128/mBio.00784-19 10.1128/mBio.00784-19

# Response

**Published:** 2019-11-01

**Authors:** Victor Chaumeau

**Affiliations:** Shoklo Malaria Research Unit, Thailand

We thank to reviewer for her useful comments on the manuscript. The wording “natural infection” was changed in the title and throughout the manuscript as per reviewer’s suggestion. In addition, part of the discussion was rewritten and suggested reference was added in order to better emphasize this study limitation in the revised version of the manuscript. The possibility of environmental contamination for all sequences including that of the supergroup B was explicitly discussed in the Discussion section. The amplicon produced in no-template controls and negative samples certainly results from the formation of primer dimers, which are unlikely to reach the size of 438 bp. Therefore, it should be possible to discriminate between primer dimers and amplification of the PCR DNA target using gel electrophoresis. Nevertheless, the reviewer is right to question the value of conventional PCR in the field of molecular diagnostic given the high risk of false positive by contamination [1]. This issue has been extensively discussed in the manuscript and we do not wish to add more details in the current version of the manuscript. Point-by-point answers to specific comments are listed below:

*1.         The information on the origin of the laboratory-reared wMel-infected A. aegypti is missing. Were they made by the authors or are they the published strains?*

*Wolbachia*-infected reference samples were kindly provided by Dr. Lauren Carrington from the Oxford University Clinical Research Unit, Ho Chi Minh City, Vietnam. Corresponding reference [2] was added in the methods section of the revised version of the manuscript.

*2.         The sequence of W16S primer, the amplicon sizes for both qPCR reactions and the rationale behind trying both primer sets should be added.*

 The sequence of W16S primer, the amplicon sizes for both qPCR reactions and the rationale behind trying both primer sets were added in the Methods section in the revised version of the manuscript.

*3.         In the discussion, p7/28, first line: the integration into the chromosome would have produced “higher CP values”, and not as it is - “lower”.*

 The integration of *Wolbachia* genome into the mosquito chromosome would have resulted in a higher copy number of PCR DNA target in the sample (several millions versus a few dozens), and therefore in a lower CP value given the negative correlation that exists between the CP value and the logarithm of the concentration of PCR DNA target in the sample.

 1.            Apfalter P, Reischl U, Hammerschlag MR. In-house nucleic acid amplification assays in research: how much quality control is needed before one can rely upon the results? J Clin Microbiol. 2005;43 12:5835-41; doi: 10.1128/jcm.43.12.5835-5841.2005. http://dx.doi.org/10.1128/jcm.43.12.5835-5841.2005.

 2.            Carrington LB, Tran BCN, Le NTH, Luong TTH, Nguyen TT, Nguyen PT, et al. Field- and clinically derived estimates of Wolbachia-mediated blocking of dengue virus transmission potential in Aedes aegypti mosquitoes. Proc Natl Acad Sci U S A. 2018;115 2:361-6; doi: 10.1073/pnas.1715788115. http://dx.doi.org/10.1073/pnas.1715788115.

# Peer review: Reviewer response for version 2

**DOI:** 10.21956/wellcomeopenres.16771.r35969

**Published:** 2019-09-06

**Authors:** Francesco Baldini

**Affiliations:** 1 Institute of Biodiversity Animal Health and Comparative Medicine, University of Glasgow, Glasgow, UK

The second version of this manuscript provides greater clarity and detailed methodological information. Limitations of the study are also discussed. Overall the manuscript contains novel insights on natural *Wolbachia *strains in *Anopheles *species from Myanmar, calling for further investigations on the role of these endosymbionts on the biology of malaria mosquitoes.

I confirm that I have read this submission and believe that I have an appropriate level of expertise to confirm that it is of an acceptable scientific standard.

# Peer review: Reviewer response for version 2

**DOI:** 10.21956/wellcomeopenres.16771.r35968

**Published:** 2019-07-22

**Authors:** Thomas Walker

**Affiliations:** 1 Department of Disease Control, London School of Hygiene & Tropical Medicine, London, UK

Figure 2 has been added to show the typical result of the qPCR asay used for *Wolbachia *detection in mosquito samples. The Wspec results (panel A) show that CP values were generated from no template controls (some below 35) and melt curve analysis was required to differentiate primer dimers from genuine target amplification. With *Wolbachia *gene qPCR I would not have expected no template control CPs to come up at below 35 cycles and this could be due to trying to amplify a 16S gene fragment >400 base pairs (the first report of this to my knowledge). According to Table 1, the threshold CP value for using the Wspec primer set appears to be between 30-33 cycles given only 7/9 of the 10 ^-5 ^dilution amplified. This would be further supported by the CP of 10 ^-6 ^(34.95 +/-1.64) not being statistically higher that to the 10 ^-5 ^dilution (34.48 +/-0.33). The authors rightly conclude that all of the samples in this study are at threshold detection (CP values >31 cycles) and the authors correctly used both melt curve analysis and sequencing to confirm this was genuine amplification. Further details of the qPCR analysis strengthens the manuscript but I’m not entirely sure how the authors estimated bacterial load. The sentence ‘Given that DNA was extracted from whole mosquitoes, it was possible to estimate the bacterial load in single mosquitoes without using a calibrator to normalize the signal’ needs further clarification, particularly given the title has changed to include reference to ‘low density strains’. The CP values would suggest low density infections but in order to make the comparison to *w*Mel in *Aedes aegypti* you would need to account for body size and/or extraction efficiency as these factors will influence *Wolbachia *CP values and therefore estimating bacterial load. Lab-reared *Aedes aegypti *adults will likely be larger in size than wild caught *Anopheles *species so that comparison is not possible without either normalising to host genes or measuring the total DNA extracted. I also would suggest a better measure of prevalence rates would have resulted from using the Wspec primer set in the conventional PCR format (Werren & Windsor, 2000 ^1^) which has been routinely used to screen mosquito populations.  

 Figure 4 shows the phylogeny of the *Wolbachia *16S gene and it appears that the sequences are quite diverse within individual species (eg. *An. maculates *appearing to have four different strains) and the same strains appear across multiple species (eg. *An. minimus* and *An. baimaii*). Although *Wolbachia *superinfections exist in mosquito species, having the same strain of *Wolbachia *(based on 16S sequences) in multiple species would need confirmation from additional gene sequencing as this seems unlikely for endosymbiotic *Wolbachia *bacteria. The discussion does now contain an explanation that no material was left for MLST due to assessing *Plasmodium *infection rates despite having 50 μL of eluted DNA. Overall the reliance on only 16S sequences (some of which appear to have identical sequences across multiple *Anopheles *species) is still problematic in my opinion and I would think a title that contains ‘genetically diverse’ is inappropriate based on sequencing of only one *Wolbachia *gene fragment.

I confirm that I have read this submission and believe that I have an appropriate level of expertise to confirm that it is of an acceptable scientific standard, however I have significant reservations, as outlined above.

## References

[1] : Wolbachia infection frequencies in insects: evidence of a global equilibrium?. *Proc Biol Sci*.2000;267(1450) : 10.1098/rspb.2000.1139 1277-85 10.1098/rspb.2000.1139 10972121PMC1690679

# Response

**Published:** 2019-09-18

**Authors:** Victor Chaumeau

**Affiliations:** Shoklo Malaria Research Unit, Thailand

We thank to reviewer for his useful comments on the manuscript. Reviewer’s concern on the comparison of *Wolbachia* DNA titers measured in wild *Anopheles *and laboratory-reared *Aedes aegypti* was addressed by removing the corresponding sentences in the Methods, Results and Discussion sections. Our data clearly demonstrate that wild *Anopheles* are infected with very low *Wolbachia* densities given the high CP values of the screened samples (>30 cycles) and the very low CP values of the reference *Aedes aegypti *samples (<19 cycles). The reference to “low-density infections” in the title is therefore valid, and does not overlook low densities as a specific feature of the strains detected in this study. We agree with reviewer’s comment on the limitation of not having additional sequence data on other markers, and regret that it was not possible to generate such data in the current study. Although it not possible to assess accurately the phylogenic relationships between different *Wolbachia* strains only with 16S rRNA sequences, our data clearly demonstrate that the *Wolbachia *strains detected in this study are genetically diverse. Therefore, we wish to keep the title of the manuscript unchanged.

# Peer review: Reviewer response for version 1

**DOI:** 10.21956/wellcomeopenres.16370.r34658

**Published:** 2019-03-04

**Authors:** Francesco Baldini

**Affiliations:** 1 Institute of Biodiversity Animal Health and Comparative Medicine, University of Glasgow, Glasgow, UK

This work identified *Wolbachia *strains in *Anopheles *species from Myanmar. To my knowledge this is the first evidence of identification of *Wolbachia* infection in *Anopheles *mosquito populations in Asia. This work opens novel questions on the potential role of these infections on the vectorial capacity of the vector host. Indeed, recently identified natural *Wolbachia* infections in *Anopheles *species in Africa have been shown to potentially influence the vectorial capacity of the infected vectors.

 The authors state that *Wolbachia *infections were identified by using quantitative PCR approach followed by sequencing of the *Wolbachia *16S gene, although some details are missing and the methodology should be clarified. Phylogenetic analysis is also not completely detailed. Specific comments to the manuscript are listed below.

**Introduction:**

 The authors state that cytoplasmic incompatibility (CI) ‘enhances the “vertical” transmission of Wolbachia’. From my knowledge this is incorrect, as CI does not directly affect “vertical” transmission, but rather increases ‘indirectly’ the fitness of the progeny of Wolbachia infected mothers.

 The authors state that ‘Shaw et al. observed a negative correlation between Wolbachia infection and the development of *P. falciparum* oocysts in naturally blood-fed females’. This is not completely correct, as in this work the authors have quantified the prevalence of *P. falciparum* in resting blood fed females inside house 5 days after collection/blood feeding, without any prior knowledge on the infectious status of the mosquitoes; thereby, the stage of parasite infection (oocyst or sporozoite) was not investigated.  

**Methods:**

 Authors indicate Shaw et al. and Gomes et al. as references for the qPCR using W-Spec primers against Wolbachia 16S. This is where I am getting confused, as Shaw et al. did not use qPCR and Gomes et al. use a different primer set for qPCR. The methodology should be clarified.

 If W-Spec primers were used, the expected product size is >400 base pairs (bp); this bp size is often too large for qPCR, as large amplicons tend to produce secondary structures during the dissociation steps, thus resulting in multiple melting peaks. If possible, it would be informative to provide more details on the optimization of this assay (in case additional reagents were added, for example) and to show the dissociation profile of the obtained amplicons, as this would enable troubleshooting of the technique if others will try to replicate the work and/or use the same methodology. Also, it should be specified how sequencing was performed, e.g. direct purification after qPCR (how?), which primer was used, etc.

 In the phylogenetic analysis the authors should state what was the sequence size used to build the tree. Novel obtained sequences should also be uploaded and their unique identifier indicate in the article.

 Statistical analysis used in Table 1-3 should be indicated (if any).

**Results:**

 The authors state the ‘we assessed species diversity, Plasmodium and Wolbachia infection rates in these Groups’. This is incorrect as Plasmodium infection rates are not shown.

 Captions in Table 1-3 are missing information on what each column indicates. Although these tables can be generally informative, I wonder if showing species diversity using pie-charts (for each species group) over imposed on the map in Figure 1 would provide a more direct illustration of the species composition and abundances of the Anopheles species in the study area.

 As indicated in the methods, the size of the sequence used for Figure 2 should be indicated. It would be informative to include the alignment use for the tree figure.

 Figure 3b shows the overall prevalence of Wolbachia in different villages without specifying the species, so I am not sure what is the purpose of illustrating the result in this way. If this is too show that some villages have higher prevalence over others this should be indicated only if statistical analysis supports it (although I doubt this is the case if species distribution is included as a variable).

 The authors state that ‘Crossing-point values ranged from 31.0 to 40.6 amplification cycles. Infected specimens were generally infected at a density close or below the limit of detection of the assay (only one sample gave 3/3 positive reactions).’ More details should be given regarding the rational for inclusion (or exclusion) of an infected/amplified sample; it is not clear to me if ‘reactions’ refers to technical replicates in the same qPCR run or in different qPCR assays. This should be described with more details. It would also be informative to normalize the quantity of the amplified Wolbachia 16S using a mosquito housekeeping gene, for example. Indeed, as ‘density [was] close or below the limit of detection of the assay’ normalization would provide information on the likelihood of false negatives in samples, as you would expect if the total DNA is very low (for example due to inefficient DNA extraction).

**Discussion:**

 The authors state that ‘Our data and African studies confirm that the occurrence of natural Wolbachia infections has been underestimated in malaria mosquitoes.’ As direct assessment of Wolbechia prevalcence on samples previously identified as uninifected was not performed here (nor in African samples) it cannot be ruled out that previous Wolbachia negative samples were not true negatives, so this work (and others) only suggest possible underestimation in previous works, as they have not directly confirmed it.

 The authors state that ‘It was not possible to study more in detail the phylogeny of Wolbachia strain detected in this study by multi locus sequence typing because of the lack of DNA extracts after the screening.’ It is not clear to me if any attempts were made at all or not. If so, please give more details on the targeted genes and discuss why these could not be amplified.

I confirm that I have read this submission and believe that I have an appropriate level of expertise to confirm that it is of an acceptable scientific standard, however I have significant reservations, as outlined above.

# Response

**Published:** 2019-07-03

**Authors:** Victor Chaumeau

**Affiliations:** Shoklo Malaria Research Unit, Thailand

We thank to the reviewer for his useful feedback on the manuscript. Additional details on the qPCR assay used for *Wolbachia *detection and on the phylogenetic analysis were added to the revised manuscript. Answer to point-specific comments are given below:

  

- **The authors state that cytoplasmic incompatibility (CI) ‘enhances the “vertical” transmission of Wolbachia’. From my knowledge this is incorrect, as CI does not directly affect “vertical” transmission, but rather increases ‘indirectly’ the fitness of the progeny of Wolbachia infected mothers.**

Reviewer’s comment has been addressed in the revised version of the manuscript.

- **The authors state that ‘Shaw et al. observed a negative correlation between Wolbachia infection and the development of *P. falciparum* oocysts in naturally blood-fed females’. This is not completely correct, as in this work the authors have quantified the prevalence of *P. falciparum* in resting blood fed females inside house 5 days after collection/blood feeding, without any prior knowledge on the infectious status of the mosquitoes; thereby, the stage of parasite infection (oocyst or sporozoite) was not investigated.**

Reviewer’s comment has been addressed in the revised version of the manuscript.

- **Authors indicate Shaw et al. and Gomes et al. as references for the qPCR using W-Spec primers against Wolbachia 16S. This is where I am getting confused, as Shaw et al. did not use qPCR and Gomes et al. use a different primer set for qPCR. The methodology should be clarified.**

In this study, we adapted an in-house real-time PCR assay with legacy primers describe previously (W-Spec forward and reverse primers). More details and appropriate reference were added in the paragraph on *Wolbachia *detection in the Methods in section.

- **If W-Spec primers were used, the expected product size is >400 base pairs (bp); this bp size is often too large for qPCR, as large amplicons tend to produce secondary structures during the dissociation steps, thus resulting in multiple melting peaks. If possible, it would be informative to provide more details on the optimization of this assay (in case additional reagents were added, for example) and to show the dissociation profile of the obtained amplicons, as this would enable troubleshooting of the technique if others will try to replicate the work and/or use the same methodology. Also, it should be specified how sequencing was performed, e.g. direct purification after qPCR (how?), which primer was used, etc.**

Additional information on the validation of the assay and sequencing of the PCR products were added to the revised version of the manuscript as per reviewer’s suggestion.

- **In the phylogenetic analysis the authors should state what was the sequence size used to build the tree. Novel obtained sequences should also be uploaded and their unique identifier indicate in the article.**

The sequence size used to build the tree was added in the figure legend in the revised version of the manuscript. Accession number are given in the method section.

- **Statistical analysis used in Table 1-3 should be indicated (if any).**

Tables 1-3 were replaced by a single table presenting human-biting rate instead of relative proportion of each mosquito species in the corresponding group. Appropriate definition of the statistics used to calculate human-biting rate are given in the Methods section.

- **The authors state the ‘we assessed species diversity, Plasmodium and Wolbachia infection rates in these Groups’. This is incorrect as Plasmodium infection rates are not shown.**

The inaccurate statement was removed from the revised version of the manuscript.

- **Captions in Table 1-3 are missing information on what each column indicates. Although these tables can be generally informative, I wonder if showing species diversity using pie-charts (for each species group) over imposed on the map in Figure 1 would provide a more direct illustration of the species composition and abundances of the Anopheles species in the study area.**

Table 1-3 were removed from the manuscript and replaced by a single table showing human-biting rate estimates collated by village and species. Given the number of study villages and diversity of *Anopheles *mosquitoes, we do not think that figuring multiple pie-charts on the map will improve the readability of the data.

- **As indicated in the methods, the size of the sequence used for Figure 2 should be indicated. It would be informative to include the alignment use for the tree figure.**

The sequence alignment was added to the revised version of the manuscript and the number of positions in the final dataset used to build the tree was added to the figure legend of the revised version of the manuscript.

- **Figure 3b shows the overall prevalence of Wolbachia in different villages without specifying the species, so I am not sure what is the purpose of illustrating the result in this way. If this is too show that some villages have higher prevalence over others this should be indicated only if statistical analysis supports it (although I doubt this is the case if species distribution is included as a variable).**

The reviewer is right to question the relevance of our prevalence data. Given the low sample size and the diversity of *Wolbachia* strains and *Anopheles *species, prevalence data were removed from the revised version of the manuscript.

- **The authors state that ‘Crossing-point values ranged from 31.0 to 40.6 amplification cycles. Infected specimens were generally infected at a density close or below the limit of detection of the assay (only one sample gave 3/3 positive reactions).’ More details should be given regarding the rational for inclusion (or exclusion) of an infected/amplified sample; it is not clear to me if ‘reactions’ refers to technical replicates in the same qPCR run or in different qPCR assays. This should be described with more details. It would also be informative to normalize the quantity of the amplified Wolbachia 16S using a mosquito housekeeping gene, for example. Indeed, as ‘density [was] close or below the limit of detection of the assay’ normalization would provide information on the likelihood of false negatives in samples, as you would expect if the total DNA is very low (for example due to inefficient DNA extraction).**

It is common that at low parasite concentration, only some replicates give a positive result because the distribution of the DNA template in the reaction tube follow a Poisson distribution (Sterkers, Varlet-Marie et al. 2010, Stahlberg and Kubista 2014, Chaumeau, Andolina et al. 2016). This observation does not challenge the validity of our results. A clear statement that some *Wolbachia *infected sample have probably been missed because bacterial density observed in *Anopheles* are close or below the limit of detection of the assay that give 95% positive reaction. Misleading interpretations on the prevalence of *Wolbachia *infection were removed given the small sample size and the possibility of false negative. 

  

- **The authors state that ‘Our data and African studies confirm that the occurrence of natural Wolbachia infections has been underestimated in malaria mosquitoes.’ As direct assessment of Wolbechia prevalcence on samples previously identified as uninifected was not performed here (nor in African samples) it cannot be ruled out that previous Wolbachia negative samples were not true negatives, so this work (and others) only suggest possible underestimation in previous works, as they have not directly confirmed it.** **The authors state that ‘It was not possible to study more in detail the phylogeny of Wolbachia strain detected in this study by multi locus sequence typing because of the lack of DNA extracts after the screening.’ It is not clear to me if any attempts were made at all or not. If so, please give more details on the targeted genes and discuss why these could not be amplified.**

The methodology used for *Wolbachia *detection in this study was described into detail in the Methods section and we did not attempt additional experiments on *Wolbachia* than that described in the manuscript. In addition to *Wolbachia *detection and molecular identification of the mosquito species, sample were also screened for *Plasmodium *infection. There was not DNA material to perform additional experiment after the screening with the W-Specf/ W-Specr primers.

# Peer review: Reviewer response for version 1

**DOI:** 10.21956/wellcomeopenres.16370.r34878

**Published:** 2019-02-28

**Authors:** Thomas Walker

**Affiliations:** 1 Department of Disease Control, London School of Hygiene & Tropical Medicine, London, UK

The authors present an interesting study in which *Wolbachia *strains were detected in *Anopheles *species from Myanmar. This study is particularly timely given the recent discoveries of natural *Wolbachia *strains in *Anopheles *malaria vectors in Sub-Saharan Africa and evidence that these natural endosymbiotic bacteria could be influencing malaria parasite infection prevalence in wild mosquito populations. The study provides evidence for *Wolbachia *infections using amplification and sequencing of the *Wolbachia *16S gene although more clarity is needed on which primer set was used given the authors report undertaking qrtPCR and sequencing of PCR products. The manuscript would be significantly improved with additional *Wolbachia *gene analysis and to provide the quantitative PCR data. This would provide more information on the *Wolbachia *strain infections being presented and allow these strains to be put into context with recent discoveries in other *Anopheles *species.

**Introduction**

 The introduction needs significant improvement in the referencing. For example, the sentence ‘In addition, *Wolbachia *can interfere with the development of some pathogens, including dengue virus’ contains a reference to a publication that only describes *Wolbachia *establishment and invasion in an *Aedes aegypti *laboratory population (not virus inhibition). 

 Furthermore, including references 5 & 6 in the context of pathogen blocking is not appropriate given this was work which was proposing to use cytoplasmic incompatibility to reduce *Culex *mosquito populations and artificial *Wolbachia*-infected mosquito lines were only established in the mid 2000s. 

 The paragraph describing natural *Wolbachia *infections in mosquitoes also needs further references ^1^^,^^2^^,^^3^.

 The final paragraph in the introduction presents the fact that Natural *Wolbachia *infections in Southeast Asian malaria vectors have not been reported. However, the authors should reference the studies in which screening of Anopheles species for Wolbachia was undertaken despite finding no evidence of natural infections ^4^.

**Methods**

 The primers used for Wolbachia detection W-Specf (CATACC TATTCGAAGGGATAG) and W-Specr (AGCTTCGAGTGAA ACCAATTC) produce a product size of 438 bp and this (to my knowledge) would not be possible or has not been reported using a qrtPCR format. The authors also reference Gomes et al. ^5^ which used a different reverse primer (5′-TTGCGGGACTTAACCCAACA-3′) that results in amplification of a smaller fragment of the 16S rRNA gene for qrtPCR. The accession numbers MK336794 - MK336806 refer to sequences with >400 bases indicating W-Specf/W-Specr was used. The authors need to clarify if W-Specf/W-Specr was used on a qrtPCR format or if both were used independently and report the differences in prevalence rates using these two primer sets.  

**Results**

 The inclusion for analysis of only what would be considered ‘primary malaria vectors’ needs more explanation if the authors overall aim was to provide evidence for natural *Wolbachia *infections in *Anopheles *species given the mosquitoes were not screened for *Plasmodium *infection.  

 Tables 1-3 provide a breakdown of the species composition collected at the different villages but I think it would be more informative to have all the different species grouped according to villages.  Currently it’s difficult to determine mosquito species prevalence on a village level.

 The statistics used in tables 1-3 don’t appear to be explained either in the manuscript methods or in the table legends. For example, I am assuming ‘n/N’ means the species/total number collected but again this would be much easier to understand if species were grouped by villages.

**Wolbachia infections**

 With reference to my previous point raised in the methods, which 16S PCR primer set and format was used to determine the prevalence rates and to generate sequences for Figure 2? 

 Figure 2 needs to have more details included such as the number of nucleotide sequences used in the analysis and the total positions in the final dataset.

 The authors provide the overall prevalence rate (13/370) and then have Figure 3 to show the individual species. Figure 3a I don’t feel is needed because plotting 1/11 (PSE) and 1/12 (DIR) seems unnecessary and could be in a table that incorporates prevalence rates by species and village.  Having an overall village prevalence rate (Fig 3b) has little biological relevance given you have variable Anopheles species containing what appears to be different Wolbachia strains based on 16S analysis.   

 A major limitation of the phylogenetic analysis (and even the prevalence rates) is only using a single *Wolbachia *gene (16S) but I appreciate that Cp values ranging from 31-40.6 are at the limit of detection. The authors should provide these 16S Cp values to allow the reader to see the variation both between technical and biological replicates.  

 Could the authors also provide the rationale for concluding that samples were positive where not all technical replicates produced positive amplification given ‘only one sample gave 3/3 positive reactions’? How do these results fit with the Minimum Information for Publication of Quantitative Real-Time PCR Experiments (MIQE) guidelines ^6^?

 Could the authors not have used another *Wolbachia *qPCR assay based on a second gene that targets a broad range of strains ^7^?

 The density comparison to laboratory-reared Aedes aegypti artificially-infected with the wMel Wolbachia strain is not particularly informative for several reasons. Firstly, quantifying Wolbachia density without accounting for mosquito body size and/or DNA extraction efficiency is problematic. Secondly, the wMel is a strain that naturally infects *Drosophila melanogaster *so a better comparison would be to natural infections in mosquito species (such as *w*Pip in *Culex quinquefasciatus *or even the *wAlbA/wAlbB strains *in *Aedes albopictus *). Therefore, I would question the inclusion of this density data given *w*Mel in *Ae. aegypti *is an artificial infection.    

**Discussion**

 The sentence ‘It was not possible to study more in detail the phylogeny of *Wolbachia* strain detected in this study by multi locus sequence typing because of the lack of DNA extracts after the screening’ needs clarification. Do the authors mean that they were unable to amplify any of the *Wolbachia *MLST genes? Did they try using degenerate primer protocols or nested PCR given the qPCR data would indicate low density infections?

 The statement “This is consistent with previous attempts to quantify *Wolbachia* in naturally infected malaria vectors" is incorrect and refers to some (An. gambiae complex) but not all species analysed in Sub-Saharan Africa ^8^. The authors should expand this discussion as the low density infections presented in this study are comparable to those strains detected in *An. gambiae *mosquitoes from Sub-Saharan Africa. Some of these studies have only resulted in 16S gene amplification and sequencing resulting in conflicting phylogenetics which appear incompatible with the traditional criteria for vertically transmitted endosymbionts (reviewed in reference 9 ^9^). The authors should provide some further discussion points on whether their results only amplifying 16S could have resulted from either 1) integration into the mosquito genome or 2) some form of contamination. However, additional *Wolbachia* gene analysis would allow more confidence in these detected strains given the high 16S qPCR Cp values are at the limit of detection.

I confirm that I have read this submission and believe that I have an appropriate level of expertise to confirm that it is of an acceptable scientific standard, however I have significant reservations, as outlined above.

## References

[1] : Diverse novel resident Wolbachia strains in Culicine mosquitoes from Madagascar. *Sci Rep*.2018;8(1) : 10.1038/s41598-018-35658-z 17456 10.1038/s41598-018-35658-z 30498246PMC6265278

[2] : Identification of Wolbachia strains in mosquito disease vectors. *PLoS One*.2012;7(11) : 10.1371/journal.pone.0049922 e49922 10.1371/journal.pone.0049922 23185484PMC3503815

[3] : wFlu: characterization and evaluation of a native Wolbachia from the mosquito Aedes fluviatilis as a potential vector control agent. *PLoS One*.2013;8(3) : 10.1371/journal.pone.0059619 e59619 10.1371/journal.pone.0059619 23555728PMC3608659

[4] : Geographic distribution of wolbachial infections in mosquitoes from Thailand. *J Invertebr Pathol*.2013;114(3) : 10.1016/j.jip.2013.04.011 337-40 10.1016/j.jip.2013.04.011 23660513

[5] : Effect of naturally occurringWolbachia inAnopheles gambiae s.l. mosquitoes from Mali onPlasmodium falciparum malaria transmission. *Proceedings of the National Academy of Sciences*.2017;114(47) : 10.1073/pnas.1716181114 12566-12571 10.1073/pnas.1716181114 PMC570333129114059

[6] : The MIQE guidelines: minimum information for publication of quantitative real-time PCR experiments. *Clin Chem*.2009;55(4) : 10.1373/clinchem.2008.112797 611-22 10.1373/clinchem.2008.112797 19246619

[7] : Broader prevalence of Wolbachia in insects including potential human disease vectors. *Bull Entomol Res*.2015;105(3) : 10.1017/S0007485315000085 305-15 10.1017/S0007485315000085 25772521

[8] : Novel Wolbachia strains in Anopheles malaria vectors from Sub-Saharan Africa. *Wellcome Open Res*.2018;3: 10.12688/wellcomeopenres.14765.2 113 10.12688/wellcomeopenres.14765.2 30483601PMC6234743

[9] : Is Anopheles gambiae a natural host of Wolbachia?. *bioRxiv*.2018; 10.1101/491449 10.1101/491449 PMC656102031186318

# Response

**Published:** 2019-07-03

**Authors:** Victor Chaumeau

**Affiliations:** Shoklo Malaria Research Unit, Thailand

We thank to the reviewer for his useful feedback on the manuscript. More details on the qPCR assay used to detect *Wolbachia* in this study were provided in the revised version of the manuscript, including the raw quantitative data as per reviewer’s suggestion. We also agree that additional *Wolbachia* genotyping would have been an important added value to the manuscript. However, there was not enough DNA material to attempt additional genotyping of the *Wolbachia *strains detected in this study given that mosquito samples were also screened for *Plasmodium *infection and identified at the species level with molecular assays.

 Response to point specific comments are listed below:

- **The introduction needs significant improvement in the referencing. For example, the sentence ‘In addition, Wolbachia can interfere with the development of some pathogens, including dengue virus’ contains a reference to a publication that only describes Wolbachia establishment and invasion in an Aedes aegypti laboratory population (not virus inhibition). Furthermore, including references 5 & 6 in the context of pathogen blocking is not appropriate given this was work which was proposing to use cytoplasmic incompatibility to reduce Culex mosquito populations and artificial Wolbachia-infected mosquito lines were only established in the mid 2000s. The paragraph describing natural Wolbachia infections in mosquitoes also needs further references. The final paragraph in the introduction presents the fact that Natural Wolbachia infections in Southeast Asian malaria vectors have not been reported. However, the authors should reference the studies in which screening of Anopheles species for Wolbachia was undertaken despite finding no evidence of natural infections.**

The referencing of the introduction was improved as per reviewer’s suggestions.

- **The primers used for Wolbachia detection W-Specf (CATACC TATTCGAAGGGATAG) and W-Specr (AGCTTCGAGTGAA ACCAATTC) produce a product size of 438 bp and this (to my knowledge) would not be possible or has not been reported using a qrtPCR format. The authors also reference Gomes et al.** **^5^** **which used a different reverse primer (5′-TTGCGGGACTTAACCCAACA-3′) that results in amplification of a smaller fragment of the 16S rRNA gene for qrtPCR. The accession numbers MK336794 - MK336806 refer to sequences with >400 bases indicating W-Specf/W-Specr was used. The authors need to clarify if W-Specf/W-Specr was used on a qrtPCR format or if both were used independently and report the differences in prevalence rates using these two primer sets.**

Amplification of fragments much longer than 438 bp with real-time PCR technology has been reported previously (Rothfuss, Gasser et al. 2010). Without *a priori* knowledge on the DNA sequences of the *Wolbachia* strains detected in this study, the primer W-Specf and W-Specr were chosen for their ability to detect most *Wolbachia* strains infecting insects and to establish phylogenic relationship among isolates (Werren and Windsor 2000). The results of additional assay optimization and serial dilution experiments with the W-Specf/W16S primers used by *Gomes et al.* were added to the revised version of the manuscript, although we did not use these primers for the screening of *Wolbachia *in field mosquito samples.

- **The inclusion for analysis of only what would be considered ‘primary malaria vectors’ needs more explanation if the authors overall aim was to provide evidence for natural *Wolbachia *infections in *Anopheles *species given the mosquitoes were not screened for *Plasmodium *infection.**

The vector status of *Anopheles* species in the Thailand-Myanmar border area has been determined previously (Somboon, Aramrattana et al. 1998, Chaumeau, Fustec et al. 2018). Primary vectors in the study area are *An. minimus s.s.* (Minimus Complex, Funestus Group), *An. maculatus s.s.*, *An. sawadwongporni *(Maculatus Group), *An. dirus s.s.* and *An. baimaii* (Dirus Complex, Leucosphyrus Group). Proper referencing was added in the revised version of the manuscript.

- **Tables 1-3 provide a breakdown of the species composition collected at the different villages but I think it would be more informative to have all the different species grouped according to villages.  Currently it’s difficult to determine mosquito species prevalence on a village level.**

The reviewer is right to question the relevance of presenting specific diversity as a proportion of a given species in the corresponding group. In the revised manuscript, human-biting rates were reported is a single table instead of the relative proportions.

- **The statistics used in tables 1-3 don’t appear to be explained either in the manuscript methods or in the table legends. For example, I am assuming ‘n/N’ means the species/total number collected but again this would be much easier to understand if species were grouped by villages.**

In the revised version of the manuscript, table 1-3 were merged in a single table and appropriate description of the statistics used in this table were added to the Methods section.

- **With reference to my previous point raised in the methods, which 16S PCR primer set and format was used to determine the prevalence rates and to generate sequences for Figure 2?**

The primer W-Specf and W-Specr were used in a real-team PCR format for both estimation of the prevalence rates and phylogenetic analysis as described in the Methods section.

- **Figure 2 needs to have more details included such as the number of nucleotide sequences used in the analysis and the total positions in the final dataset.**

More details were added in the revised phylogenetic tree and the total number of position in the final dataset was stated in the figure legend.

- **The authors provide the overall prevalence rate (13/370) and then have Figure 3 to show the individual species. Figure 3a I don’t feel is needed because plotting 1/11 (PSE) and 1/12 (DIR) seems unnecessary and could be in a table that incorporates prevalence rates by species and village.  Having an overall village prevalence rate (Fig 3b) has little biological relevance given you have variable Anopheles species containing what appears to be different Wolbachia strains based on 16S analysis.**

The reviewer is right to question the biological significance of plotting prevalence estimates per species and per village. In the revised manuscript, the screening results collated by village and species are presented in a table, and the two plots were removed.

- **A major limitation of the phylogenetic analysis (and even the prevalence rates) is only using a single *Wolbachia *gene (16S) but I appreciate that Cp values ranging from 31-40.6 are at the limit of detection. The authors should provide these 16S Cp values to allow the reader to see the variation both between technical and biological replicates.**

We agree with the reviewer the analyzing only 16S ssuRNA genes is a limitation of our study. Raw quantitative data were added to the revised version of the manuscript as per reviewer suggestion.

- **Could the authors also provide the rationale for concluding that samples were positive where not all technical replicates produced positive amplification given ‘only one sample gave 3/3 positive reactions’? How do these results fit with the Minimum Information for Publication of Quantitative Real-Time PCR Experiments (MIQE) guidelines ?**

It is common that at low parasite concentration, only some replicates give a positive result because the distribution of the DNA template in the reaction tube follow a Poisson distribution (Sterkers, Varlet-Marie et al. 2010, Stahlberg and Kubista 2014, Chaumeau, Andolina et al. 2016). This observation does not challenge the validity of our results. A clear statement that some *Wolbachia *infected sample have probably been missed because bacterial density observed in *Anopheles* are close or below the limit of detection of the assay that give 95% positive reaction. Misleading interpretations on the prevalence of *Wolbachia *infection were removed given the small sample size and the possibility of false negative.

- **Could the authors not have used another *Wolbachia *qPCR assay based on a second gene that targets a broad range of strains.**

In the absence of *a priori *knowledge on the *Wolbachia *strains infecting *Anopheles * samples, the W-Specf/ W-Specr primers were chosen for their ability to detect a broad range of strains infecting insects (Werren and Windsor 2000). There was not enough DNA material remaining after the screening with the W-Specf/ W-Specr primers to use another assay. However, the specificity of the PCR was confirmed in all positive sample by Sanger sequencing of the PCR product.

- **The density comparison to laboratory-reared Aedes aegypti artificially-infected with the wMel Wolbachia strain is not particularly informative for several reasons. Firstly, quantifying Wolbachia density without accounting for mosquito body size and/or DNA extraction efficiency is problematic. Secondly, the wMel is a strain that naturally infects *Drosophila melanogaster *so a better comparison would be to natural infections in mosquito species (such as *w*Pip in *Culex quinquefasciatus *or even the *wAlbA/wAlbB strains *in *Aedes albopictus *). Therefore, I would question the inclusion of this density data given *w*Mel in *Ae. aegypti *is an artificial infection.**

We agree with the reviewer that there is little biological relevance in comparing the density of *Wolbachia *infection in artificially infected *Aedes aegypti *and naturally infected *Anopheles*. We think that presenting those quantitative data is an added value to support that natural *Wolbachia* infection in this study actually occur at low density rather than resulting from of a low assay sensitivity. We believe that a calibrator to normalize the signal is not necessary as DNA was extracted from whole mosquitoes and bacterial loads expressed as an (arbitrary) number of bacteria per mosquito rather the a number of bacteria per weight-unit of mosquito body (Varlet-Marie, Sterkers et al. 2014, Chaumeau, Andolina et al. 2016).

- **The sentence ‘It was not possible to study more in detail the phylogeny of *Wolbachia* strain detected in this study by multi locus sequence typing because of the lack of DNA extracts after the screening’ needs clarification. Do the authors mean that they were unable to amplify any of the *Wolbachia *MLST genes? Did they try using degenerate primer protocols or nested PCR given the qPCR data would indicate low density infections?**

The methodology used for *Wolbachia *detection in this study was described into detail in the Methods section and we did not attempt additional experiments on *Wolbachia* than that described in the manuscript. In addition to *Wolbachia *detection and molecular identification of the mosquito species, samples were also screened for *Plasmodium *infection (data not shown). There was no DNA material to perform additional experiment after the screening with the W-Specf/ W-Specr primers.

- **The statement “This is consistent with previous attempts to quantify *Wolbachia* in naturally infected malaria vectors" is incorrect and refers to some (An. gambiae complex) but not all species analysed in Sub-Saharan Africa** **^8^** **. The authors should expand this discussion as the low density infections presented in this study are comparable to those strains detected in *An. gambiae *mosquitoes from Sub-Saharan Africa. Some of these studies have only resulted in 16S gene amplification and sequencing resulting in conflicting phylogenetics which appear incompatible with the traditional criteria for vertically transmitted endosymbionts (reviewed in reference 9** **^9^** **). The authors should provide some further discussion points on whether their results only amplifying 16S could have resulted from either 1) integration into the mosquito genome or 2) some form of contamination. However, additional *Wolbachia* gene analysis would allow more confidence in these detected strains given the high 16S qPCR Cp values are at the limit of detection.**

We thank to the reviewer for his useful feedback on the interpretation of our quantitative data. Suggested edits and references were added in the revised version of the manuscript.

**References**

 Chaumeau, V., C. Andolina, B. Fustec, N. Tuikue Ndam, C. Brengues, S. Herder, D. Cerqueira, T. Chareonviriyaphap, F. Nosten and V. Corbel (2016). "Comparison of the Performances of Five Primer Sets for the Detection and Quantification of Plasmodium in Anopheline Vectors by Real-Time PCR." PLoS One **11**(7): e0159160.

 Chaumeau, V., B. Fustec, S. Nay Hsel, C. Montazeau, S. Naw Nyo, S. Metaane, S. Sawasdichai, P. Kittiphanakun, P. Phatharakokordbun, N. Kwansomboon, C. Andolina, D. Cerqueira, T. Chareonviriyaphap, F. H. Nosten and V. Corbel (2018). "Entomological determinants of malaria transmission in Kayin state, Eastern Myanmar: A 24-month longitudinal study in four villages." Wellcome Open Research **3**(109).

 Rothfuss, O., T. Gasser and N. Patenge (2010). "Analysis of differential DNA damage in the mitochondrial genome employing a semi-long run real-time PCR approach." Nucleic Acids Res **38**(4): e24.

 Somboon, P., A. Aramrattana, J. Lines and R. Webber (1998). "Entomological and epidemiological investigations of malaria transmission in relation to population movements in forest areas of north-west Thailand." Southeast Asian J Trop Med Public Health **29**(1): 3-9.

 Stahlberg, A. and M. Kubista (2014). "The workflow of single-cell expression profiling using quantitative real-time PCR." Expert Rev Mol Diagn **14**(3): 323-331.

 Sterkers, Y., E. Varlet-Marie, S. Cassaing, M. P. Brenier-Pinchart, S. Brun, F. Dalle, L. Delhaes, D. Filisetti, H. Pelloux, H. Yera and P. Bastien (2010). "Multicentric comparative analytical performance study for molecular detection of low amounts of Toxoplasma gondii from simulated specimens." J Clin Microbiol **48**(9): 3216-3222.

 Varlet-Marie, E., Y. Sterkers, M. P. Brenier-Pinchart, S. Cassaing, F. Dalle, L. Delhaes, D. Filisetti, H. Pelloux, F. Touafek, H. Yera and P. Bastien (2014). "Characterization and multicentric validation of a common standard for Toxoplasma gondii detection using nucleic acid amplification assays." J Clin Microbiol **52**(11): 3952-3959.

 Werren, J. H. and D. M. Windsor (2000). "Wolbachia infection frequencies in insects: evidence of a global equilibrium?" Proc Biol Sci **267**(1450): 1277-1285.
